# Efficacy of artificial saliva in preventing oral changes in patients admitted to the ICU: a controlled clinical trial

**DOI:** 10.1007/s00784-026-06864-1

**Published:** 2026-04-24

**Authors:** Edmundo Duarte Martins, Nayara Isabelle Cabral Rebouças, Sophia Queiroz Marques dos Santos, Tatiana Bernardo Farias Pereira, Rosângela Oliveira da Câmara, Maria do Rosário Avelino Bezerra Silva, Alzira Maria Batista Guará, Ana Clara Guedes Queiroz, Addison Ribeiro de Almeida, Leandro De Santis Ferreira, Ericka Janine Dantas da Silveira

**Affiliations:** 1 Department of Dentistry, Federal University of Rio Grande do Norte - UFRN, Av. Senador Salgado Filho 1787, Natal, Rio Grande do Norte 59056-000 Brazil; 2 General João Machado Hospital, Natal, RN Brazil; 3 Department of Chemistry, Federal University of Rio Grande do Norte- UFRN, Natal, Rio Grande do Norte Brazil

**Keywords:** Intensive care units, Saliva, artificial, Xerostomia, Mouth mucosa

## Abstract

**Objective:**

To evaluate the effect of prophylactic artificial saliva application on the prevention of oral changes in patients admitted to the Intensive Care Unit (ICU).

**Materials and methods:**

This controlled clinical trial was conducted in an ICU between October 2024 and May 2025. Patients were assigned to an Experimental Group (EG), which received topical artificial saliva after standard oral hygiene, and a Control Group (CG), which underwent oral hygiene only. Statistical analyses were performed using Jamovi, SPSS, and Stata. Fisher’s exact test, Kaplan–Meier survival analysis with log-rank test, and Poisson regression were applied, adopting a significance level of *p* < 0.05.

**Results:**

Among the 46 patients analyzed (21 in EG and 25 in CG), oral changes occurred in 61% during hospitalization. The Control Group developed significantly more lesions than the Experimental Group (*p* = 0.023), showing a 29% higher occurrence of changes. Oral candidiasis (24.1%) and dry mouth (20.2%) were more frequent in the CG, whereas traumatic ulceration predominated in the EG (51.6%).

**Conclusion:**

The prophylactic use of artificial saliva was associated with a lower occurrence of oral changes in critically ill patients and may represent a simple and potentially cost-effective supportive intervention in the ICU setting.

**Clinical relevance:**

The findings suggest that prophylactic use of artificial saliva may contribute to reducing oral complications in critically ill patients. As a simple and low-cost intervention, it has the potential to support oral care strategies in ICU settings; however, further studies are needed to confirm its effectiveness and inform clinical protocols.

## Introduction

 The Intensive Care Unit (ICU) is a specialized hospital setting designed to deliver comprehensive and continuous care to patients with life-threatening conditions or those requiring close physiological monitoring and advanced medical support [1, 2]. Nevertheless, the ICU environment and the invasive procedures commonly performed in this context can adversely influence patients’ physiological stability, homeostatic balance, and overall comfort [3]. Consequently, oral health can be compromised due to the patients’ overall medical condition and the complexity of the treatments administered. Several factors commonly observed in critically ill patients such as systemic diseases, continuous medication use, advanced age, and therapeutic interventions may contribute to reduced salivary flow. The association between chronic conditions, long-term medication use, and hyposalivation has been widely reported in the literature.

Reduced salivary flow (hyposalivation) and the sensation of dry mouth (xerostomia) can significantly impair overall health, and both conditions contribute to the development of oral diseases such as dental caries, periodontal disease, candidiasis, mucosal ulcerations, and other opportunistic infections [4–7]. In critically ill patients, decreased oral motility further aggravates hyposalivation and xerostomia, since saliva production depends not only on gustatory and olfactory stimuli but also on mechanical stimulation [8]. Furthermore, ICU patients who require various life-support devices, such as an orotracheal tube, often remain with their mouth open or partially open for extended periods, which also promotes mucosal dehydration [9, 10].

Thus, the essential functions of saliva, such as lubrication and humidification, maintenance of oral homeostasis, antimicrobial protection, digestion, food bolus formation and clearance, taste perception and retronasal olfaction, buffering capacity, mineralization, and wound healing [11–13], may be compromised in ICU patients who experience xerostomia or hyposalivation. Furthermore, oral dryness may lead to considerable discomfort, as thirst is frequently reported as an intense and distressing symptom among ICU patients [14]. In this context, different strategies can be employed to reduce the complications associated with reduced salivary flow, depending on the severity of hyposalivation. Although mechanical or pharmacological salivary stimulation is considered the first-line approach in patients with preserved glandular responsiveness [15], ICU patients are often too debilitated to cooperate with such methods [5, 16].

Therefore, artificial saliva may serve as an alternative to minimize the symptoms of oral dryness and improve patients’ quality of life [12]. By incorporating synthetic agents, this product may reduce the risk of pressure injuries caused by friction between therapeutic devices and dehydrated mucosa [17]. Artificial saliva can also support the natural lubricating functions of saliva, particularly in patients with mechanical damage to soft and hard oral tissues [11].

Considering the clinical relevance of maintaining adequate oral homeostasis in critically ill patients and the lack of established strategies to manage hospital-acquired hyposalivation, this issue becomes particularly important in the Intensive Care Unit (ICU). Hyposalivation is a prevalent condition among ICU patients [18], capable of causing significant discomfort and strongly associated with other oral changes, yet it is frequently overlooked or left untreated [19]. Therefore, this study aimed to evaluate the efficacy of artificial saliva in preventing the development of oral changes during ICU hospitalization, in order to better understand its potential benefits and clinical applications.

## Methods

### Study design and patients

**Fig. 1:**
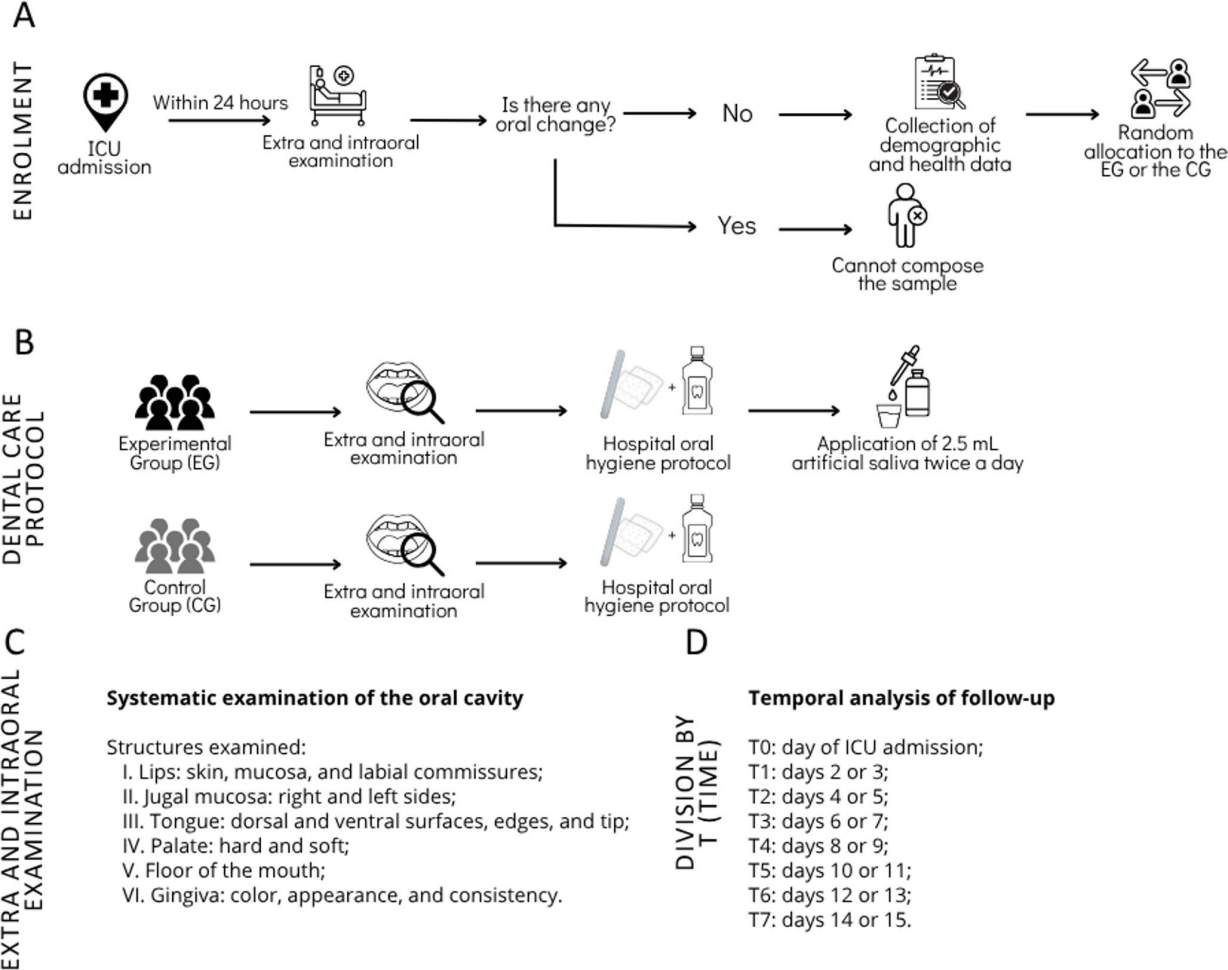
Methodological aspects of the study. (**A**) Diagram of the patient inclusion; (**B**) Procedures performed in each patient group; (**C**) Sequence of steps for the oral physical examination; (**D**) Details of the time points for patient assessment and data collection during the oral examination

This single-blind controlled clinical trial was conducted in the Intensive Care Unit (ICU) of Hospital Geral Dr. João Machado, Natal/RN, Brazil. Participants were allocated to either the Experimental Group (EG) or the Control Group (CG) according to the order of ICU admission using a sequential alternating allocation method (Fig. 1A). Each newly admitted eligible patient was alternately assigned to one of the two groups. This allocation strategy was adopted due to the consecutive inclusion of patients and the operational characteristics of the ICU setting, where patient enrollment occurred in real time and the number of simultaneously eligible participants was limited. Allocation was performed by the researcher responsible for patient enrollment. The study was designed as single-blind, with the outcome assessor blinded to group allocation. The study protocol was prospectively registered in the Brazilian Clinical Trials Registry (ReBEC; RBR-10fqpzdg), and the clinical phase was conducted from October 2024 to May 2025.

Data collected included demographic variables (age and sex), type of respiratory support, and general and oral health status. Inclusion criteria comprised patients aged ≥ 18 years, of either sex, who underwent dental evaluation within the first 24 h of ICU admission, presented no oral or perioral alterations at baseline, and were fully dependent on professional oral care. Exclusion criteria included a history of autoimmune disease, pregnancy, known hypersensitivity to any component of the gel, inability to undergo intraoral examination (e.g., limited mouth opening), or prior ICU admission at another institution. Patients who remained in the ICU for less than 48 h were also excluded.

The primary outcome was the development of oral alterations during the follow-up period. The unit of analysis was the patient-day, allowing the assessment of temporal changes in oral conditions in a repeated-measures context.

Sample size calculation was performed based on the comparison of two independent proportions. The estimated proportion of success (prevention of oral alterations) in the control group was assumed to be 49% (P₁ = 0.49), according to Martins et al. [5]. For the EG, it was assumed that the use of artificial saliva would increase the prevention rate by 35%, resulting in an expected proportion of 84% (P₂ = 0.84). Considering a significance level of 5% (α = 0.05; Zα/2 = 1.96) and a statistical power of 80% (1–β = 0.80; Z1–β = 0.84), the minimum required sample size was estimated at 25 participants per group, totaling 50 patients. To account for potential losses or exclusions during follow-up, a 15% increase was applied, resulting in a target sample size of 29 participants per group.

Patients in the EG received 2.5 mL of artificial saliva gel every 12 h following the standard oral hygiene protocol. The CG underwent only routine oral hygiene. The sequence of procedures is presented in Fig. 1B; Table 1. The daily applications were performed by trained dental or nursing staff previously instructed on the study protocol. All batches of the artificial saliva gel were prepared by the same local pharmacy (Table 2).

**Table 1:** Sequence of the care protocol for the experimental group (EG)

Step	Procedure
01	Put on non-sterile procedure gloves
02	Measure 2.5 mL of the gel using a graduated cup
03	Put on sterile surgical gloves
04	Perform the standard oral hygiene protocol routinely adopted by the service: oral hygiene using gauze wrapped around a wooden spatula, with distilled water and 0.12% chlorhexidine digluconate, using posterior-to-anterior scraping movements
05	Apply the salivary substitute to the entire oral mucosa in a posterior-to-anterior direction, using a gloved finger, as follows: tongue, floor of the mouth, buccal mucosa, palate, and the buccal, lingual, and occlusal surfaces of the teeth
06	Aspirate any excess gel from the oropharynx at the end of the procedure

**Table 2:** Formulation of the Artificial Saliva

Compound	Proportion
Anhydrous citric acid	0.2%
Purified water q.s.p. or fluid gel q.s.p.	100mL
Sodium bicarbonate	2%
Carboxymethylcellulose (CMC)	0.8 g
Calcium chloride	0.012 g
Magnesium chloride	0.004 g
Potassium chloride	0.096 g
Sodium chloride	0.067 g
Mint flavoring essence	0.5%
Sodium fluoride	2ppm
Potassium phosphate	0.027 g
Methylparaben (Nipagin)	0.001 g
Propylparaben (Nipazol)	0.01 g
Xylitol	2.4 g

#### Blinding and data collection

This study was conducted with blinded outcome assessors. Patients admitted to the ICU were under sedation during the study period and therefore were not aware of their allocation to the study groups. However, the clinicians responsible for the baseline evaluation and participant allocation were not blinded.

Data collection was conducted in two stages. Initially, two dentists from the hospital’s multidisciplinary team performed a standardized bedside clinical examination prior to participant allocation to the control and experimental groups. The intraoral and extraoral physical examination was performed between 8:00 and 10:00 a.m., using visual inspection and palpation with the aid of flashlights, wooden spatulas, and gauze, in order to establish the patients’ baseline oral condition. These same professionals were also responsible for allocating participants to the study groups.

A second group of dentists, blinded to group allocation (EG or CG), was responsible for data collection and clinical evaluation of the patients during the intervention period. Information regarding each patient’s oral and systemic conditions was recorded in their medical records and in standardized research forms, including documentation of the artificial saliva application.

**Fig. 2:**
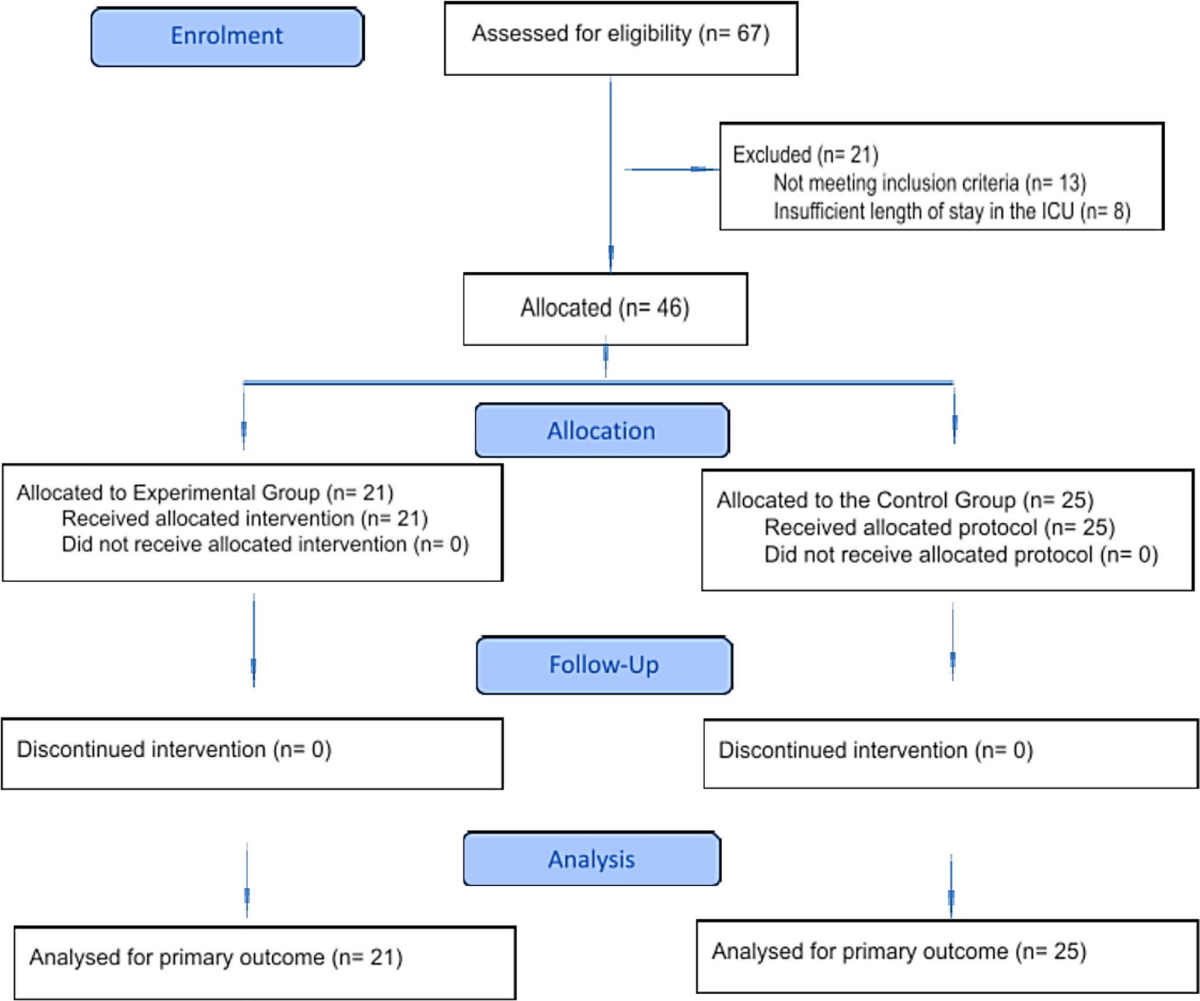
Flowchart of patient recruitment, allocation, follow‑up

Oral alterations were assessed through systematic extra- and intraoral examinations (Fig. 1C), based on the criteria described by Martins et al. [5], including: (1) angular cheilitis; (2) oral pseudomembranous candidiasis; (3) coated tongue; (4) oral tissue dryness; (5) ulceration; (6) petechiae; (7) lingual atrophy; (8) gingival inflammation; (9) hairy tongue; and (10) spontaneous bleeding during oral manipulation. Patients were followed prospectively from ICU admission until discharge, death, transfer to another unit, or recovery of the ability to perform independent oral hygiene. Follow-up assessments were conducted at predefined intervals (T1–T7), as illustrated in Fig. 1D, considering the earliest day assessed within each interval. Figure 2 presents the flowchart describing the processes of patient recruitment, group allocation, and follow-up throughout the study.

All examiners underwent prior training and calibration conducted by a specialist. Inter-examiner agreement was assessed using the Kappa statistic, which indicated fair agreement (κ = 0.40; *p* < 0.001).

### Physicochemical evaluation of the artificial saliva

The stability of the artificial saliva was assessed at the Quality Control Laboratory of the Department of Chemistry, UFRN (Natal, Rio Grande do Norte, Brazil). Spectrophotometric and pH analyses were performed using a UV-Vis spectrophotometer (GENESYS 180, Thermo Scientific, Waltham, USA) and a digital pH meter (PHS-3E, Even, Shanghai, China), respectively. Optical stability was evaluated by measuring the absorbance of the saliva substitute at a wavelength of 600 nanometers (nm) using the UV-Vis spectrophotometer, selected for its sensitivity to turbidity associated with precipitation, chemical reactions, or microbial proliferation. Acid-base stability was monitored by measuring the pH using a properly calibrated pH meter. In both tests, samples were prepared in triplicate, stored under controlled conditions, and analyzed at 0, 12, 24, and 48 h, since artificial saliva has a limited permanence in the oral cavity due to the solubility, ingestion, and/or absorption of the compound, which is why reapplication was performed every 12 h.

**Fig. 3:**
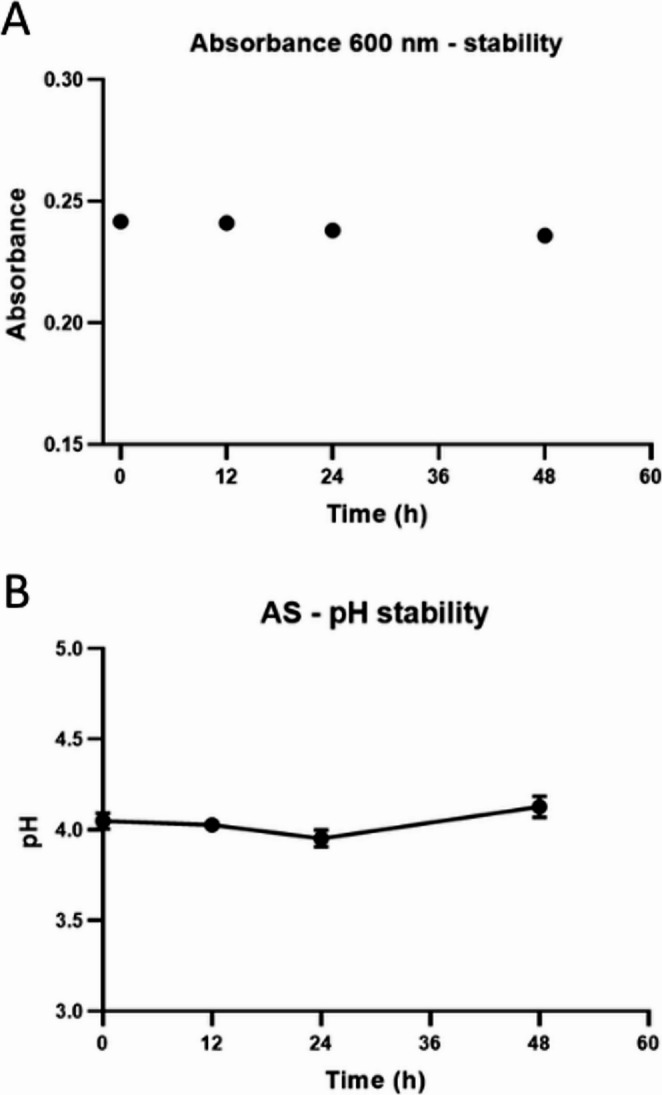
Physicochemical parameters of the artificial saliva formulation. (**A**) Absorbance values at 600 nm; (**B**) pH measurements across the analysis intervals

The analyses demonstrated that the saliva substitute remained stable throughout the 48-hour evaluation period. The initial absorbance at 600 nm was 0.242, decreasing slightly to 0.236 at the end of the experiment, without any variations suggestive of physicochemical instability or turbidity formation attributable to chemical reactions or microbial proliferation (Fig. 3A). Additionally, the pH remained around 4.0 throughout all analysis intervals, with values ranging from 3.9 to 4.17 (Fig. 3B), corresponding to fluctuations of less than 0.2 units, which are considered acceptable for liquid formulations. Therefore, the two evaluated parameters confirm the maintenance of optical homogeneity and the acid–base stability of the saliva substitute during the observation period.

### Statistical analysis

Data were organized using Microsoft Excel (Office 365, Microsoft Corporation, Redmond, USA) and exported to Jamovi (The jamovi project, version 2.4), IBM SPSS Statistics (Statistical Package for the Social Sciences for Windows, version 20.0), and Stata (Stata Statistical Software: Release 14) for statistical analysis. The chi-square test was initially used to compare the overall frequency of oral alterations between the two groups. Fisher’s exact test was applied when appropriate to assess associations between evaluation days, the presence or absence of oral alterations, and the study groups. Time-to-event analyses were performed using the Kaplan–Meier method to estimate the probability of remaining free of oral alterations during follow-up, and survival curves were compared using the log-rank test. Poisson regression was used to analyze the prevalence of oral alterations throughout the follow-up period. Exploratory Cox proportional hazards regression analyses were also performed to evaluate the potential influence of individual characteristics and lesion types as confounding factors. However, due to the relatively small sample size and limited number of events, these multivariable models produced unstable estimates and were not retained in the final analyses. A significance level of *p* < 0.05 was adopted for all analyses.

## Results

### Patient profile

A total of 46 patients were included in this study, with 21 allocated to the EG and 25 to the CG (Fig. 2). The overall mean length of stay in the ICU was 10.1 days (median: 6.0).

The characteristics of patients in both groups are summarized in Table 3. In the EG (*n* = 21), 13 patients (61.9%) were male and 8 (38.1%) were female, with a mean age of 66.4 years (median: 66.0; standard deviation: 12.0). The CG included 25 patients, of whom 21 (84.0%) were male and 4 (16.0%) were female, presenting a mean age of 66.1 years (median: 66.0; standard deviation: 17.3).

**Table 3:** Sample profile

Variable	Control group (*N* = 25)		Experimental group (*N* = 21)	
	*N*	%	*N*	%
Sex				
Male	21	84.0	13	61.90
Female	4	16.0	8	38.10
Age (years)				
25–61	8	32.0	6	28.57
62–75	8	32.0	9	42.86
76–97	9	36.0	6	28.57
Respiratory support				
Room air	18	72.0	9	42.86
Oro tracheal intubation	7	28.0	12	57.14
Underlying disease				
Acute myocardial infarction (AMI)	12	48.0	7	33.32
Renal disease	0	0.0	3	14.29
Cardiac disease	1	4.0	1	4.76
Vascular disease	2	8.0	0	0.0
Sepsis	2	8.0	3	14.29
Pulmonary disease	0	0.0	3	14.29
Exogenous intoxication	1	4.0	0	0.0
Pneumonia	3	12.0	0	0.0
Bacterial infection	0	0.0	1	4.76
Stroke	1	4.0	0	0.0
Confidential data*	3	12.0	3	14.29

*It was not possible to access the underlying disease information for 6 patients, as this data was kept confidential

### Artificial saliva was associated with a lower occurrence of oral changes in ICU patients

**Fig. 4:**
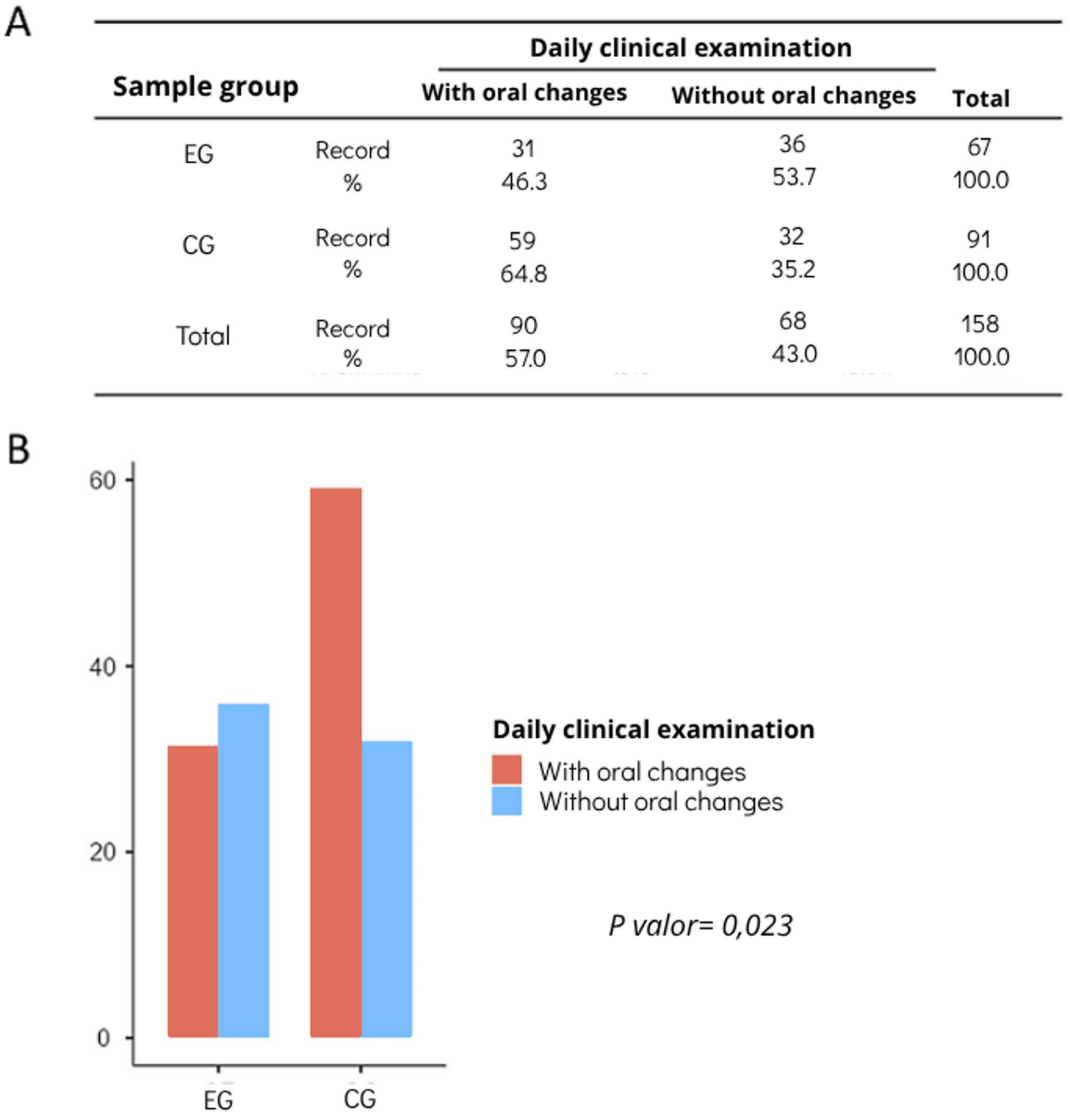
Occurrence of oral alterations during the follow-up period in the Control Group (CG) and Experimental Group (EG). (**A**) Comparative distribution of the number of oral alterations developed over the evaluation period between CG and EG. (**B**) Bar graph showing the association between the use of artificial saliva and the occurrence of oral alterations, demonstrating a significantly lower frequency of oral alterations in the EG compared with the CG (*p* < 0.05)

Among patients in the CG, 64% developed at least one oral change during the observation period, while in the EG this proportion was 57.1%. The overall prevalence of oral alterations in the sample was 60.8%. When the days evaluated were considered as the unit of analysis, the proportion of days with oral changes was higher in the CG (64.8%) compared to the EG (46.3%) (Figure 4A). In this approach, the use of artificial saliva was associated with a lower occurrence of days with oral alterations (RR = 0.714; 95% CI: 0.529–0.962; p = 0.023), suggesting a relative reduction of approximately 29% per day evaluated (Figure 4B).

**Fig. 5:**
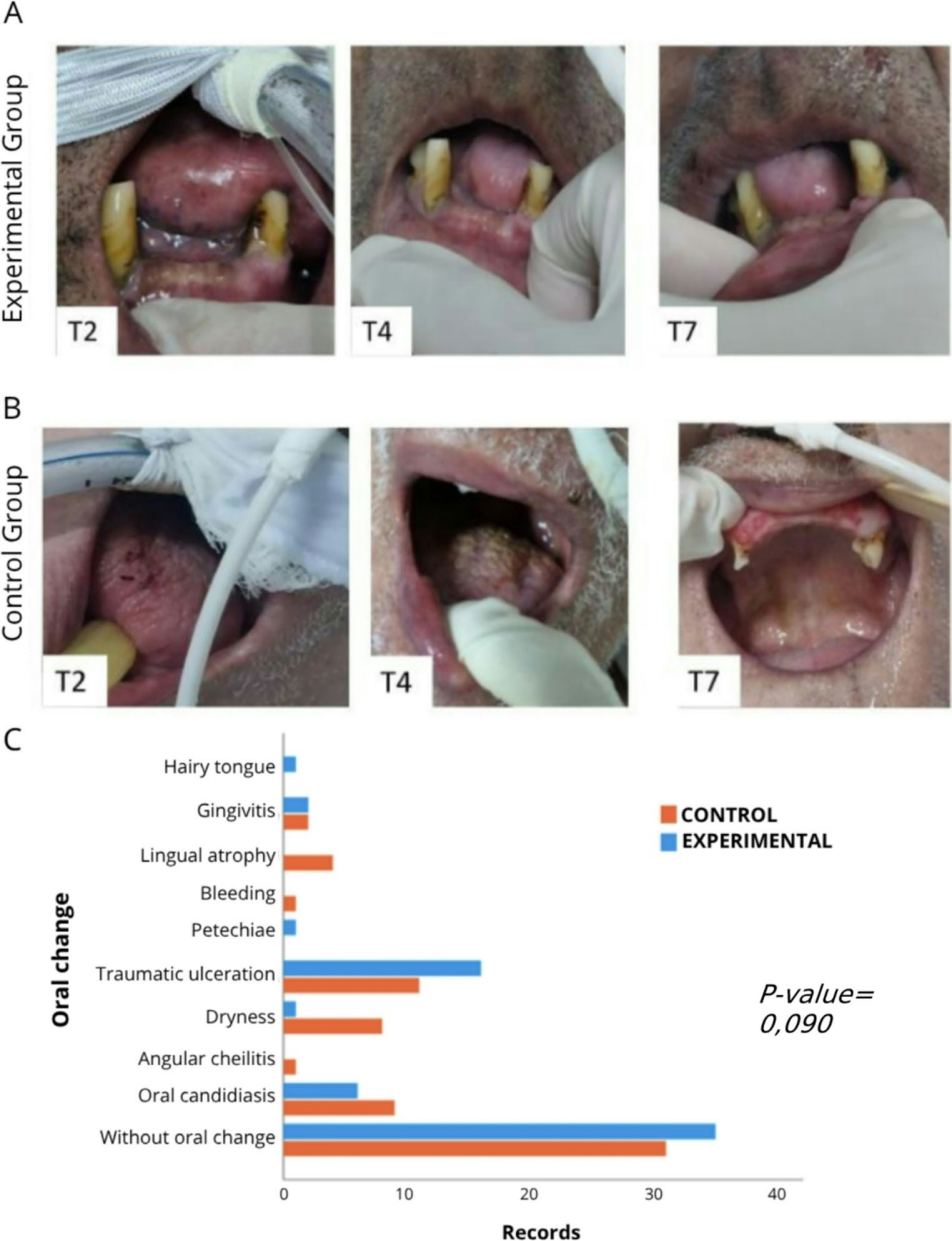
Relative risk of oral alterations associated with the use of artificial saliva and representative clinical findings during follow-up. (**A**, **B**) Representative clinical images from the CG and EG, respectively. (**C**) Distribution of the frequency and types of oral alterations identified throughout the follow-up period

Figure5 illustrates representative clinical examples from both study groups (A and B), as well as the frequency and types of oral alterations identified throughout the follow-up period (C), considering the days evaluated (clinical records) as the unit of analysis.

Considering that each patient contributed multiple observations over time, Poisson regression was used to estimate the prevalence ratio adjusted for repeated measures. In this analysis, the GE group presented a PR = 0.843 (95% CI: 0.749–2.032), with a wide confidence interval and including unity, indicating uncertainty in the estimate and a lack of robust statistical evidence of effect. This value suggests a 16% reduction in the occurrence of oral alterations in the EG.

**Fig. 6:**
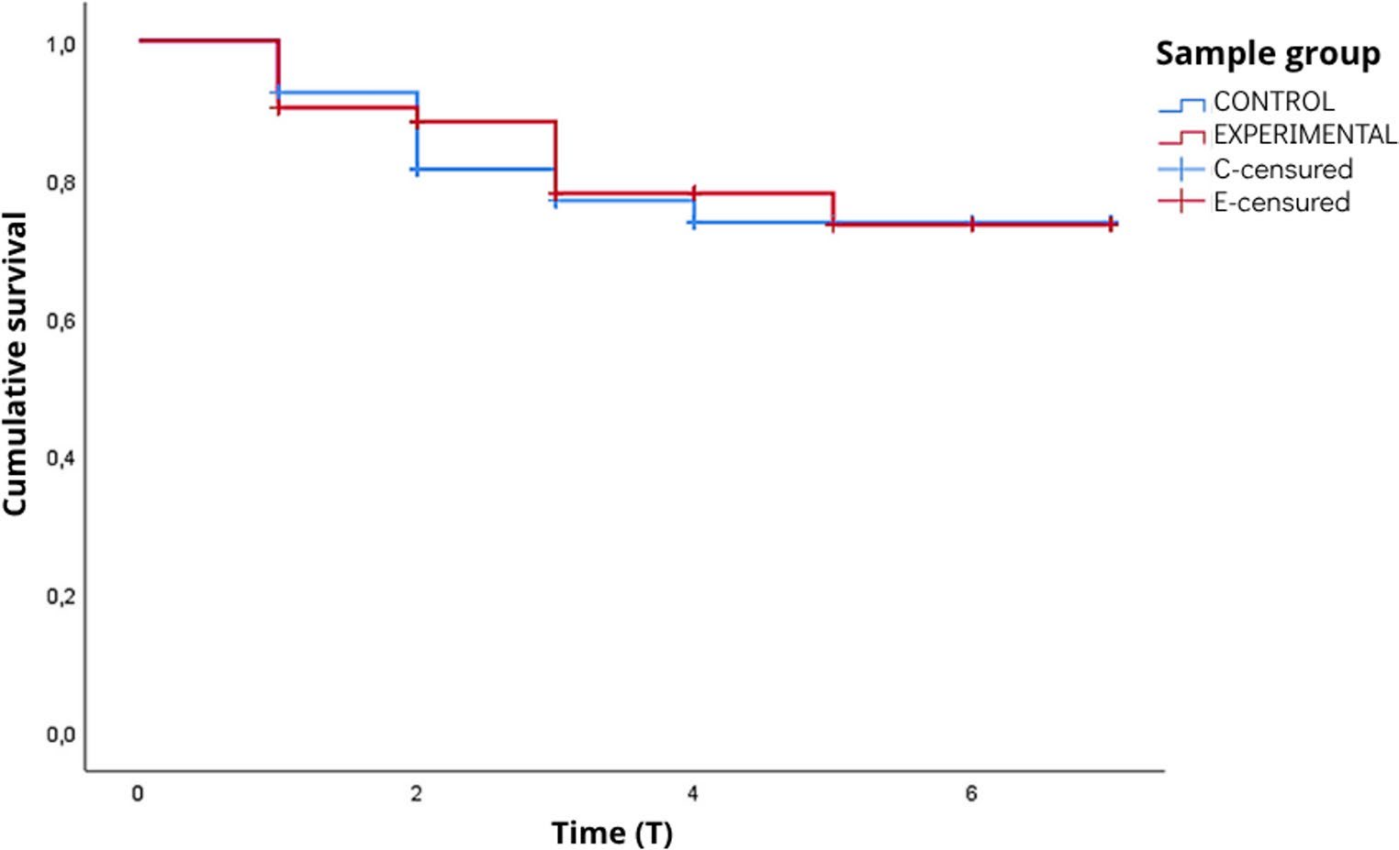
Kaplan–Meier curves of the probability of remaining free of oral alterations over time in the Control Group (CG) and Experimental Group (EG) (log-rank test, *p* = 0.825)

The survival analysis (Kaplan-Meier), which considered the time to the first event per patient, showed similar trajectories between the groups (log-rank test *p* = 0.825). Table 4 presents the survival analysis considering the time until the first occurrence of oral alteration, using the patient as the unit of analysis and, in each interval, the number of individuals at risk to calculate the survival function and respective confidence intervals. This difference between approaches can be explained by the fact that the analysis by days evaluated considers recurrent events over time, while the survival analysis considers only the first event, reducing the number of events analyzed and, consequently, the statistical power. When comparing the probability of remaining free of oral alterations between the groups, both curves followed similar trajectories, with gradual declines throughout follow-up (Fig. 6).

**Table 4:** Survival function for the occurrence of oral lesions according to the study group, illustrating the incidence of lesions over time throughout the study period

Time (interval)	Total	Events	Survival function	95% CI
1	33	11	0.915	(0.890–0.940)
2	25	7	0.848	(0.815–0.885)
3	22	5	0.775	(0.733–0.817)
4	12	1	0.759	(0.715–0.803)
5	13	1	0.737	(0.689–0.785)
6	9	0	-	-
7	9	0	-	-

Overall, although the analysis by days evaluated suggests a possible protective effect of artificial saliva, the patient-level analyses and the wide confidence intervals indicate that the results should be interpreted with caution.

## Discussion

Saliva is indispensable for maintaining oral health and is also essential for overall human health due to its fundamental role in preserving the balance of the oral environment by protecting against infections, lubricating oral tissues, and assisting in wound healing processes [20]. These functions become even more critical in settings such as ICU, where patients, particularly those receiving mechanical ventilatory support may experience impaired salivary production as a result of multiple factors, including a partially open mouth, the use of specific medications, polypharmacy, and compromised systemic conditions [18].

Salivary deficiency can lead to serious complications, such as increased risk of oral infections, dryness and fissures that predispose to traumatic lesions, and biofilm accumulation [21, 22]. To our knowledge, this is the first study conducted in critically ill patients to demonstrate that the use of artificial saliva can may contribute to the protection of oral tissues during hospitalization, highlighting its potential relevance as a simple and feasible strategy to minimize oral complications in the ICU setting.

Although artificial saliva substitutes cannot replicate the enzymatic-digestive and viscoadhesive functions of natural saliva, they should ideally match its biophysical properties, including lubrication. In our research, physicochemical analysis revealed that the tested artificial saliva formulation showed high stability, with minimal fluctuation both in absorbance at 600 nm and in pH, with minimal variations over 48 h. These findings indicate that the formulation’s structural and chemical integrity was preserved for the duration of the study. Such stability is a crucial positive attribute, as it supports proper preservation, storage, and potential clinical efficacy by ensuring product safety and predictability during extended use.

However, it should be noted that the formulation presented a pH around 4.0, which is lower than the physiological pH of natural saliva. Although acidic formulations may raise concerns regarding potential effects on enamel or oral mucosa during prolonged exposure, in the present study the product was used for a short period and under clinical supervision, and no adverse effects were observed. Future studies may consider optimizing the formulation to achieve a pH closer to physiological saliva while maintaining its physicochemical stability and stimulatory properties.

A significant frequency of oral alterations was observed among patients admitted to the ICU in this study. However, this frequency, on analysis days, was lower in patients who received artificial saliva daily, with an approximate 29% reduction in the occurrence of oral alterations and about a 16% lower chance of developing these alterations. These findings suggest that, in terms of follow-up days, the use of artificial saliva was associated with a lower frequency of days with recorded oral alterations. According to Martins et al. [5], patients hospitalized in ICU exhibit a high probability of developing oral alterations, primarily hyposalivation and oral candidiasis.

Our research showed that in the EG, 57.1% of patients were receiving orotracheal intubation as respiratory support, a proportion higher than that observed in the control group. This baseline difference should be considered when interpreting the results, as it may represent a potential confounding factor, given that orotracheal intubation is known to increase the risk of oral complications. Interestingly, despite the higher proportion of intubated patients in the EG who might theoretically present a greater susceptibility to oral alterationsthe group showed a lower overall frequency of oral lesions, with traumatic ulcerations predominating (51.6%).

Orotracheal intubation is a major cause of such traumatic lesions [21], particularly in the context of reduced salivary flow. Consequently, patients admitted to the ICU are at increased risk of pressure injuries resulting from direct and prolonged contact between medical devices and the oral tissues, especially in situations of limited mobility [23]. This scenario heightens susceptibility to persistent lesions, as continuous friction between the mucosa and the devices, combined with reduced salivary flow, may exacerbate tissue damage [5]. In the present study, the use of artificial saliva likely had a limited effect on these specific outcomes, as the salivary substitute would need to be applied directly beneath the device to create an effective lubricating barrier capable of reducing friction between the tube and adjacent tissues.

Meanwhile, the CG presented a higher frequency of candidiasis and xerostomia, consistente with other studies that reported a high prevalence of oral candidiasis in similar groups [18, 24, 25]. These findings may suggest that the intervention applied to the EG is associated with a lower predisposition to the development of infectious and dryness-related lesions, although studies with larger samples are necessary to confirm this hypothesis.

Regarding the profile of this study, the patients were mostly male, representing 84% of the CG and 61.9% of the EG. The mean age was similar between the groups, around 66 years, indicating a predominance of an elderly population, an age group more susceptible to severe systemic conditions and in-hospital complications [5]. These characteristics are consistent with a demographic study based on the analysis of 15,202 medical records, which reported a predominance of male patients (60.4%) and a mean age of 61 years among ICU admissions [26]. In this context, it is worth highlighting the high prevalence of xerostomia in the elderly population, particularly in patients with diabetes, chronic diseases, and those on long-term medication [27, 28]. Regarding underlying diseases, there was a predominance of cardiovascular conditions in both groups, especially. Acute myocardial infarction was the most frequent, accounting for 33.3% of cases in the GE and 48.0% in the CG. These clinical variables are inherent to the ICU setting and can independently influence the oral outcomes assessed; therefore, these differences should be considered when interpreting the results, since no multivariate adjustment was performed to control for covariates.

Another relevant finding observed was the survival analysis, in which, when comparing the time to lesion onset in the control and experimental groups, similar times were observed until the appearance of the first oral alteration. Although, no significant statistical difference between the groups (*p* = 0.825), which may be attributed, at least in part, to the limited sample size and the small number of events observed during follow-up, when analysis of the trend over time revealed that the group that received artificial saliva exhibited a slightly more favorable curve, suggesting a possible protective effect. This behavior may indicate that, in a longer follow-up or in a larger sample size, the intervention may have greater potential to exert more evident clinical benefits. This finding supports the hypothesis that artificial saliva may contribute to reducing the incidence of oral alterations in critically ill patients admitted to the ICU. Similarly, the result of the study by Assery [29] reported that the intervention with artificial saliva successfully improved patients’ oral health and quality of life. Thus, artificial saliva proves to be a valuable alternative for moisturizing the oral mucosa, preventing pressure injuries, accelerating wound healing, and alleviating the sensation of dry mouth [30].

Thirst and dry mouth are common issues in the ICU and are strongly associated with physical discomfort [9, 31]. Among patients with chronic systemic diseases, the use of specific medications has been identified as a contributing factor to reduced salivary flow [32]. This reduction may negatively affect patient comfort and quality of life througth symptoms such as dry mouth, viscous saliva, halitosis, and difficulty using dental prostheses [18]. Therefore, the perception of thirst should be a concern for the healthcare team, as relieving this symptom is an important component of humanized care. Doi et al. reported that afollowing oral care, all 86 patients in the sample remained free of thirst immediately after the intervention, 70 after 1 h, 29 after 2 h, 10 after 3 h, and 9 after 4 h [33]. In this sense, besides promoting tissue lubrication, artificial saliva can help reduce the sensation of dry mouth and improve comfort during speech and swallowing. These benefits go beyond the clinical aspect, as the relief of uncomfortable symptoms is directly related to the preservation of dignity in critically ill patients, who are often in a state of extreme vulnerability due to their health condition.

The use of artificial saliva should be incorporated into a comprehensive and multidisciplinary care strategy. As with any therapeutic intervention, its prescription requires an accurate diagnosis and careful consideration of the patient’s individual needs. Moreover, attention must be given to the multiple, multifactorial risk factors that contribute to oral dryness and that may not be adequately addressed by the use of salivary substitutes alone. Artificial saliva primarily acts by promoting lubrication and moisturizing of the oral mucosa, helping to reduce friction, facilitate oral function, and maintain mucosal integrity in situations of reduced salivary flow. These properties are largely related to the rheological characteristics of the formulation, which aim to reproduce the viscoelastic behavior of natural saliva and support the protective functions of the salivary film [11, 12].

Another relevant aspect in this study concerns the development of artificial saliva and the appropriate adjustment of its rheological properties to those of natural saliva, aiming to approximate the viscosity parameters of physiological saliva [12]. This approach is particularly important because natural saliva plays a key role in maintaining oral homeostasis, contributing to lubrication, tissue protection, and the balance of the oral environment [13]. However, the formulation proposed here should be used exclusively under the same conditions and objectives in which it was tested, namely in a hospital setting, for short-term use, and under professional supervision.

This study has some limitations that should be considered when interpreting its findings. The relatively small sample size and the short follow-up period may not have been sufficient to capture long-term effects of the intervention or the full manifestation of late oral complications. Previous studies have also reported short ICU lengths of stay (mean of approximately 4 days), which represents a challenge for clinical investigations requiring prolonged observation [33]. Another limitation relates to the allocation procedure. Due to the limited number of simultaneously eligible patients and the real-time nature of recruitment in the ICU setting, conventional randomization methods could not be implemented. Participants were therefore assigned using a sequential alternating allocation strategy, which does not ensure allocation concealment and may introduce potential selection bias.

Additionally, the clinical heterogeneity inherent to critically ill patients and the difficulty in controlling for all systemic factors that may influence oral conditions in the ICU environment may have affected the observed outcomes. We acknowledge that the final sample size was smaller than initially planned, which may have reduced the statistical power to detect small differences between groups and increased the possibility of type II error. Therefore, the findings should be interpreted with caution, particularly regarding analyses involving a limited number of events. Another limitation relates to the reliability of the clinical assessments. The inter-examiner agreement obtained (Kappa = 0.40) indicates a fair level of agreement. Although prior training and calibration of clinical criteria were conducted before data collection, this result suggests the possibility of variability in the assessment of oral alterations, which may have influenced the consistency of the recorded outcomes.

Nevertheless, when considered collectively, the findings of the present study suggest that artificial saliva may play a relevant role as a preventive strategy, although the observed effects were limited. The favorable trend observed highlights the need for further studies, particularly well-designed multicenter randomized controlled trialls with longer follow-up periods and adequately powered sample sizes, to confirm the clinical efficacy of this intervention and to determine whether the observed reduction in lesion rates is sustained across different clinical settings.

Moreover, this study explores an area that remains insufficiently investigated in the literature, as the interface between intensive care, saliva, and oral health, providing evidence that may contribute to the development and implementation of feasible and low-cost care protocols for the management of oral dryness in critically ill patients. Addicionally, cost-effectiveness analyses are encouraged, especially considering the hospital setting and the proposed low-cost formulations, allowing for the evaluation of the economic viability of the intervention on a large scale.

In conclusion, the findings of this study suggest that the prophylactic use of artificial saliva in ICU patients was associated with a lower occurrence of oral alterations during the follow-up period, beacuse a lower frequency of specific conditions, such as oral candidiasis and mucosal dryness, was observed in the EG. As a simple and low-cost intervention, it has the potential to support oral care strategies in ICU settings, particularly as an adjunct to routine oral hygiene measures aimed at maintaining mucosal hydration and reducing oral dryness. However, our findings should be interpreted with caution, and further studies with larger sample sizes and longer follow-up periods are needed to confirm this potential protective effect.

## Data Availability

The datasets generated and/or analyzed during this study are available from the corresponding author upon reasonable request.
